# From intervention to interventional system: towards greater theorization in population health intervention research

**DOI:** 10.1186/s12889-019-6663-y

**Published:** 2019-03-25

**Authors:** Linda Cambon, Philippe Terral, François Alla

**Affiliations:** 1 Chaire Prévention, ISPED, Université Bordeaux, Bordeaux, France; 2 Université Bordeaux, CHU, Inserm, Bordeaux Population Health Research Center, UMR 1219, CIC-EC 1401, Bordeaux, France; 3 Université Paul Sabatier, Toulouse 3, CRESCO EA 7419 - F2SMH, Toulouse, France

**Keywords:** Intervention, Public health, Intervention research, Theory, System, Complexity

## Abstract

**Background:**

Population health intervention research raises major conceptual and methodological issues. These require us to clarify what an intervention is and how best to address it.

This paper aims to clarify the concepts of intervention and context and to propose a way to consider their interactions in evaluation studies, especially by addressing the mechanisms and using the theory-driven evaluation methodology.

**Main text:**

This article synthesizes the notions of intervention and context. It suggests that we consider an “interventional system”, defined as a set of interrelated human and non-human contextual agents within spatial and temporal boundaries generating mechanistic configurations – mechanisms – which are prerequisites for change in health. The evaluation focal point is no longer the interventional ingredients taken separately from the context, but rather mechanisms that punctuate the process of change. It encourages a move towards theorization in evaluation designs, in order to analyze the interventional system more effectively. More particularly, it promotes theory-driven evaluation, either alone or combined with experimental designs.

**Conclusion:**

Considering the intervention system, hybridizing paradigms in a process of theorization within evaluation designs, including different scientific disciplines, practitioners and intervention beneficiaries, may allow researchers a better understanding of what is being investigated and enable them to design the most appropriate methods and modalities for characterizing the interventional system. Evaluation methodologies should therefore be repositioned in relation to one another with regard to a new definition of “evidence”, repositioning practitioners’ expertise, qualitative paradigms and experimental questions in order to address the intervention system more profoundly.

## Background

Population health intervention research has been defined as “the use of scientific methods to produce knowledge about policy and program interventions that operate within or outside of the health sector and have the potential to impact health at the population level” [1] (see Table 1). This research raises a number of conceptual and methodological issues concerning, among other things, the interaction between context and intervention. This paper therefore aims to synthesize these issues, to clarify the concepts of intervention and context and to propose a way of considering their interactions in evaluation studies, especially by addressing the mechanisms and using the theory-driven evaluation methodology.

**Table 1:** Definitions of terms used

Label	Definition	Sources
Intervention research	The use of scientific methods to produce knowledge about policy and program interventions that operate within or outside of the health sector and have the potential to impact health at the population level	Hawe, 2009 [1]
Intervention	A series of inter-related events occurring within a system where the change in outcome (attenuated or amplified) is not proportional to the change in input. Interventions are thus considered as ongoing social processes rather than fixed and bounded entities	Hawe et al., 2009 [11]
Context	Spatial and temporal conjunction of events, individuals and social interactions generating causal mechanisms that interact with the intervention and possibly modifying its outcomes by	Poland, Frohlich and Cargo, 2008 [4]
Mechanism	Entities and activities organized such that they are productive of regular changes from start or set-up to finish or termination of conditions	Machamer et al [14]
	An element of reasoning and reaction of an agent with regard to an intervention productive of an outcome in a given context	Ridde et al. 2012 [16]; Lacouture 2015 [15]
	The processes by which a behavior change technique regulates behavior	Michie et al. 2013 [8]
Interventional system	A set of interrelated human and non-human contextual agents within spatial and temporal boundaries generating mechanistic configurations – mechanisms – which are prerequisites for change in health	This article
Classic theory	Theories that originate from fields external to implementation science, e.g. psychology, sociology and organizational theory, which can be applied to provide understanding and/or explanation of aspects of implementation	Nilsen 2015 [31]
Implementation theory	Theories that have been developed by implementation researchers (from scratch or by adapting existing theories and concepts) to provide understanding and/or explanation of aspects of implementation	Nilsen 2015 [31]

## Main text

### To clarify the notions of intervention, context and system

#### What is an intervention?

According to the International Classification of Health Interventions (ICHI), “a health intervention is an act performed for, with or on behalf of a person or population whose purpose is to assess, improve, maintain, promote or modify health, functioning or health conditions” [2]. Behind this simple definition lurks genuine complexity, creating a number of challenges for the investigators circumscribing, evaluating and transferring these interventions. This complexity arises in particular from the strong influence of what is called the context [3], defined as a “spatial and temporal conjunction of events, individuals and social interactions generating causal mechanisms that interact with the intervention and possibly modifying its outcomes” [4]. Acknowledgement of the influence of context has led to increased interest in process evaluation, such as that described in the Medical Research Council (MRC) guideline [5]. It defines the complexity of intervention by pinpointing its constituent parts. It also stresses the need for evaluations “to consider the influence of context insofar as it affects how we understand the problem and the system, informs intervention design, shapes implementation, interacts with interventions and moderates outcomes”.

#### Intervention components

How should intervention and context be defined when assessing their specificities and interactions? The components of the interventions have been addressed in different ways. Some authors have introduced the concept of “intervention components” [6] and others that of “active ingredients” [7, 8] as a way to characterize interventions more effectively and distinguish them from context. For Hawe [9], certain basic elements of an intervention should be examined as a priority because they are “key” to producing an effect. She distinguishes an intervention’s theoretical processes (“key functions”) that must remain intact and transferable, from the aspects of the intervention that are structural and contingent on context. Further, she and her colleagues introduced a more systemic approach to intervention [10, 11]. Intervention could be defined as “a series of inter-related events occurring within a system where the change in outcome (attenuated or amplified) is not proportional to change in input. Interventions are thus considered as ongoing social processes rather than fixed and bounded entities” [11]. Both intervention and context are thus defined as being dynamic over time, and interact with each other.

#### The notion of mechanisms

To understand these interactions between context and intervention, we can use the work by Pawson and Tilley [12] on realistic evaluation. This involves analyzing the configurations between contextual parameters, mechanisms and outcomes (CMO). As such, we can consider the process of change as being marked by various intermediate states illustrated by mechanisms.

Mechanisms may be the result of a combination of factors which can be human (knowledge, attitudes, representations, psychosocial and technical skills, etc.) or material (called “non-human” by Akrich et al. [13]). The notion of mechanism has various definitions. Some authors, such as Machamer et al. [14]*,* define them as “entities and activities organized such that they are productive of regular changes from start or set-up to finish or termination of conditions”. Others define them more as prerequisites to outcomes, as in the realistic approach: a mechanism is “an element of reasoning and reaction of an agent with regard to an intervention productive of an outcome in a given context” [15, 16]. They can be defined in health psychology as “the processes by which a behavior change technique regulates behavior” [8]. This could include, for instance, how practitioners perceive an intervention’s usefulness, or how individuals perceive their ability to change their behavior.

Due to the combinations of contextual and interventional components, the process of change therefore produces mechanisms, which in turn produce effects (final and intermediate outcomes). For instance, we could consider that a motivational interview for smoking cessation could produce different psychosocial mechanisms, such as motivation, perception of the usefulness of cessation and self-efficacy. These mechanisms influence smoking cessation. This constitutes causal chains, defined here as the way in which an ordered sequence of events in the chain causes the next event. These mechanisms may also affect their own contextual or interventional components as a system. For example, the feeling of self-efficacy could influence the choice of smoking cessation supports.

#### From the intervention to the interventional system

Because the mechanism is the result of the interaction between the intervention and its context, the line between intervention and context becomes blurred [17]. Thus, rather than intervention, we suggest using “interventional system”, which includes interventional and contextual components. An interventional system is produced by successive changes over a given period in a given setting.

In this case, mechanisms become key to understanding the interventional system and could generally be defined as “what characterizes and punctuates the process of change and hence, the production of outcomes”. As an illustration, they could be psychological (motivation, self-efficacy, self-control, skills, etc) in behavioral intervention or social (values shared in a community, power sharing perception, etc.) in socio-ecological intervention.

**Fig. 1:**
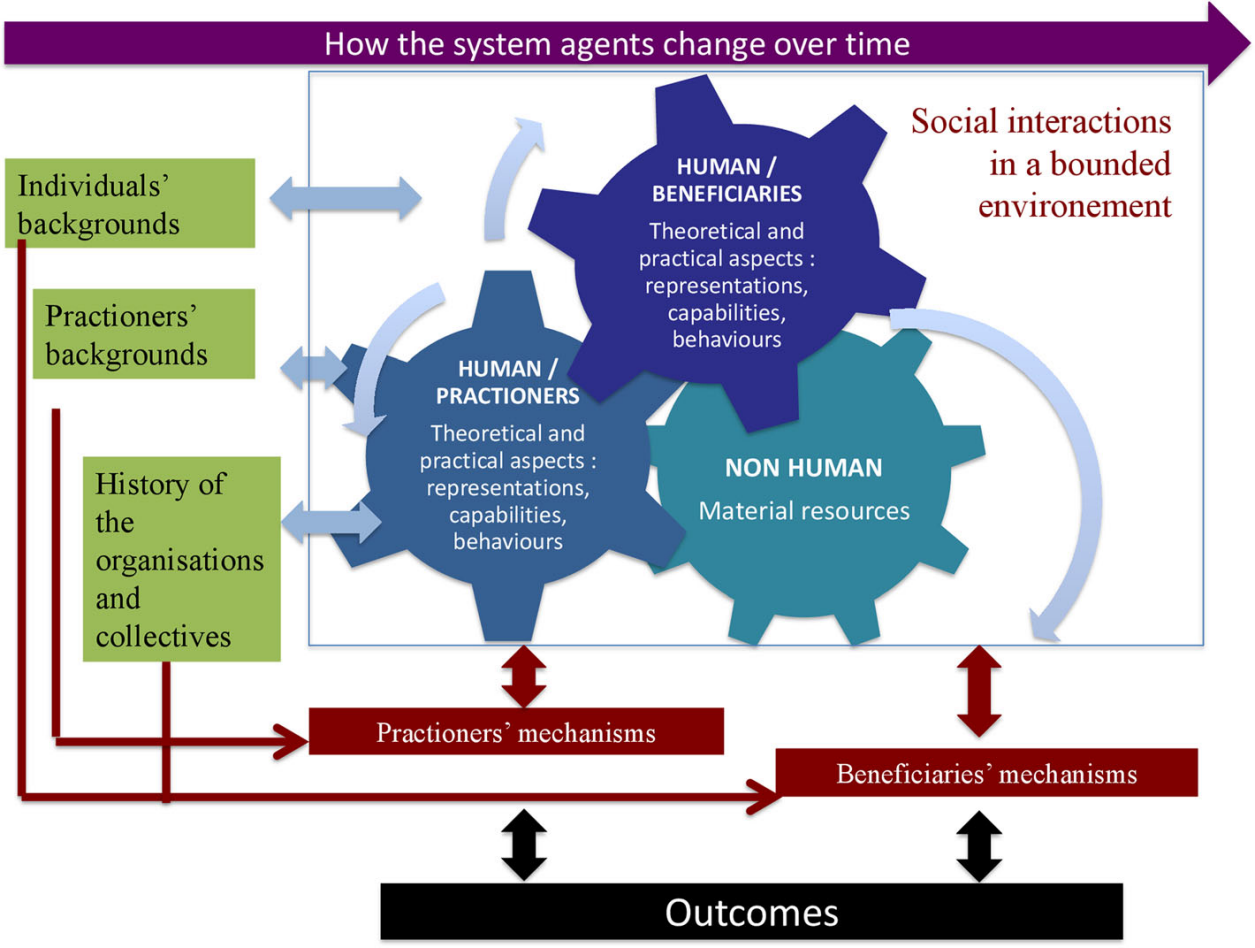
The interventional system

In light of the above, we propose to define the interventional system in population health intervention research as: A set of interrelated human and non-human contextual agents within spatial and temporal boundaries generating mechanistic configurations – mechanisms – which are prerequisites for change in health**.** In the same way, we could also consider that the intervention could in fact be an arrangement of pre-existing contextual parameters influencing their own change over time. Figure 1 illustrates this interventional system.

### Combining methods to explore the system’s key mechanisms

#### Attribution versus contribution: a need for theorization

The dynamic nature of interventional systems raises the question of how best to address them in evaluation processes. Public health has historically favored research designs with strong internal validity [18], based on experimental designs. Individual randomized controlled trials are the gold standard for achieving causal attribution by counterfactual comparison in an experimental situation. Beyond the ethical, technical or legal constraints known in population health intervention research [19], trials in this field have a major drawback: they are “blind” to the contextual elements which do influence outcomes, however. Their theoretical efficacy may well be demonstrated, but their transferability is weak, which becomes an issue as intervention research is supposed to inform policy and practice [20]. Breslow [22] made the following statement: “Counterfactual causality with its paradigm, randomization, is the ultimate black box.” However, the black box has to be opened in order to understand how an intervention is effective and how it may be transferred elsewhere.

More in line with the notion of the interventional system, other models depart completely from causal attribution by counterfactual methods. They use a contributive understanding of an intervention through mechanistic interpretation, focusing on the exploration of causal chains [23]. In other words, instead of “does the intervention work? ” the question becomes “given the number of parameters influencing the result (including the intervention components), how did the intervention meaningfully contribute to the result observed?” This new paradigm promotes theory-driven evaluations (TDE) [24, 25], which could clarify intervention-contextual configurations and mechanisms. In TDEs, the configurations and mechanisms are hypothesized by combining scientific evidence and the expertise of practitioners and researchers. The hypothetical system is then tested empirically. If this is conclusive, evidence therefore exists of contribution, and causal inferences can be made. Two main categories of TDEs can be distinguished [24, 26]: realist evaluation and theories of change.

#### Realistic evaluation

In the first one, developed by Pawson and Tilley [12], intervention effectiveness depends on the underlying mechanisms at play within a given context. The evaluation consists in identifying context-mechanism-outcome configurations (CMOs), and their recurrences are observed in successive case studies or in mixed protocols, such a realist trials [27]. The aim is to understand how and under what circumstances an intervention works. In this approach, context is studied with and as a part of the intervention. This moves us towards the idea of an interventional system. For example, we applied this approach to the “Transfert de Connaissances en REGion” project (TC-REG project), an evaluation of a knowledge transfer scheme to improve policy making and practices in a health promotion and disease prevention setting in French regions [28]. This protocol describes the way in which we combined evidence and stakeholders’ expertise in order to define an explanatory theory. This explanatory theory (itself based on a combination of sociological and psychological classic theories) hypothesizes mechanism-context configurations for evidence-based decision-making. The three steps to build the theory in the TC-REG project [28] are: step 1/ a literature review of evidence-based strategies of knowledge transfer and mechanisms to enhance evidence-based decision making (e.g. the perceived usefulness of scientific evidence); step 2 / a seminar with decision makers and practitioners to choose the strategies to be implemented and hypothesize the mechanisms potentially activated by them, along with any contextual factors potentially influencing them (e.g. the availability of scientific data.) 3/ a seminar with the same stakeholders to elaborate the theory combining strategies, contextual factors and mechanisms to be activated. The theory is the interpretative framework for defining strategies, their implementation, the expected outcomes and all the investigation methods.

#### Theory of change

In theory of change [25, 29, 30], the intervention components or ingredients mentioned earlier are fleshed out and examined separately from those of context, as a way to study how they contribute to producing outcomes. As with realistic evaluation, the initial hypothesis (the theory) is based on empirical assumptions (i.e. from earlier evaluations) or theoretical assumptions (i.e. from social or psychosocial theories). What is validated (or not) is the extent to which the explanatory theory, including implementation parameters (unlike realist evaluation), corresponds to observations: expected change (i.e. 30 mins of daily physical activity); presence of individual or socio-ecological prerequisites for success (i.e. access to appropriate facilities, sufficient physical ability, knowledge about the meaning of physical activity, etc.) based on psychosocial or organizational theories (e.g. social cognitive theory, health belief model) called classic theories [31]; effectivity of actions to achieve the prerequisites for change (i.e. types of intervention or necessary environmental modifications and their effects) based on implementation theories [31] (e.g COM-B model: Capacity-Opportunity-Motvation – Behaviour Model).; effectivity of actions conducive to these prerequisites (i.e. use of the necessary intellectual, human, financial and organizational (…) resources). This can all be mapped out in a chart for checking [30]. Then, the contribution of the external factors of the intervention to the outcomes can be evaluated. For an interventional system, in both categories, the core elements to be characterized in TDE would be the mechanisms as prerequisites to outcome. The identification of these mechanisms should confirm the causal inference, rather than demonstrating causal attribution by comparison. By replicating these mechanisms, the interventions can be transferred [21, 32]. In the case of TDEs, interventional research can be developed by natural experiment [33], allowing mechanisms to be explored, in order to explain the causal inferences, in a system which is outside the control of investigators. The GoveRnance for Equity ENvironment and Health in the City (GREENH-City) project illustrates this. It aims to address the conditions in which green areas could contribute to reducing health inequality by intervening on individual, political, organizational or geographical factors [34]. The researchers combined evidence, theories, frameworks and multidisciplinary expertise to hypothesize the potential action mechanisms of green areas on health inequalities. The investigation plans to verify these mechanisms by a retrospective study via qualitative interviews. The final goal is to determine recurring mechanisms and conditions for success by cross-sectional analysis and make recommendations for towns wishing to use green areas to help reduce health inequality.

In addition, new statistical models are emerging in epidemiology. They encourage researchers to devote more attention to causal modelling. [35].

#### The intervention theory

For both methods, before intervention and evaluation designs are elaborated, sources of scientific, theoretical and empirical knowledge should be combined to produce the explanatory theory (with varying numbers of implementation parameters). We call this explanatory theory the “intervention theory” to distinguish it from classic generalist psychosocial, organizational or social implementation theories, determinant frameworks or action models [31], which can fuel the intervention theory. The intervention theory would link activities, mechanisms (prerequisites of outcomes), outcomes and contextual parameters in causal hypotheses.

Note that to establish the theory, the contribution of social and human sciences (e.g. sociology, psychology, history, anthropology) is necessary. For example, the psychosocial, social and organizational theories enable investigators to hypothesize and confirm many components, mechanisms and their relationships involved in behavioral or organizational interventions. In this respect, intervention research becomes subordinate to the hybridization of different disciplines.

#### Combination of theory-based approaches and counterfactual designs

Notwithstanding the epistemic debates [36], counterfactual designs and theory-based approaches are not opposed, but complementary. They answer different questions and can be used successively or combined during an evaluation process. More particularly, TDEs could be used in experimental design, as some authors suggest [27, 36–38]. This combination provides a way of comparing data across evaluations; in sites which have employed both an experimental design (true control group) and theory-based evaluation, an evaluator might, for example, look at the extent to which the success of the experimental group hinged upon the manipulation of components identified by the theory as relevant to learning.

On this basis, both intervention and evaluation could be designed better. For example, the “Évaluation de l’Efficacité de l’application Tabac Info service” (EE-TIS) project [39] combines a randomized trial with a theory-based analysis of mechanisms (motivation, self-efficacy, self-regulation, etc.) which are brought about through behavioral techniques used in an application for smoking cessation. The aim is to figure out how the application works, which techniques are used by users, which mechanisms are activated and for whom. Indeed in EE-TIS project [39], we attributed one or several behavioral change techniques [8] to each feature of the “TIS” application (messages, activities, questionnaires) and identified three mechanisms– potentially activated by them and supporting smoking cessation (i.e. motivation, self-efficacy, knowledge). This was carried out by a multidisciplinary committee in 3 steps: step 1/ two groups of researchers attributed behavior change techniques to each feature, step 2/ both groups compared their results and drew a consensus and step 3/ researchers presented their results to the committee which will in turn draw a consensus. To validate these hypotheses, a multivariate analysis embedded into the randomized control trial will make it possible to figure out which techniques influence which mechanisms and which contextual factors could moderate these links.

Other examples exist which combine a realist approach and trial designs [27, 38].

#### Interdisciplinarity and stakeholder involvement

A focal point in theorizing evaluation designs is the interdisciplinary dimension, especially drawing on the expertise of social and human sciences and of practitioners and intervention beneficiaries [40]. As an intervention forms part of and influences contextual elements to produce an outcome, the expertise and feedback of stakeholders, including direct beneficiaries, offers valuable insights into how the intervention may be bringing about change. In addition, this empowers stakeholders and promotes a democratic process, which is to be upheld in population health [40]. The theorization could be done through specific workshops, including researchers, practitioners and beneficiaries on an equal basis. For example, the TC-REG project [28] has held a seminar involving both prevention practitioners and researchers, the aim being to discuss literature results and different theories/frameworks in order to define the explanatory theory (with context-mechanism configurations) and intervention strategies to be planned to test it.

## Conclusion

Population health intervention research raises major conceptual and methodological issues. These imply clarifying what an intervention is and how best to address it. This involves a paradigm shift in order to consider that in intervention research, intervention is not a separate entity from context, but rather that there is an interventional system that is different from the sum of its parts, even though each part does need to be studied in itself. This gives rise to two challenges. The first is to integrate the notion of the interventional system, which underlines the fact that the boundaries between intervention and context are blurred. The evaluation focal point is no longer the interventional ingredients taken separately from their context, but rather mechanisms punctuating the process of change, considered as key factors in the intervention system. The second challenge, resulting from the first, is to move towards a theorization within evaluation designs, in order to analyze the interventional system more effectively. This would allow researchers a better understanding of what is being investigated and enable them to design the most appropriate methods and modalities for characterizing the interventional system. Evaluation methodologies should therefore be repositioned in relation to one another with regard to a new definition of “evidence”, including the points of view of various disciplines, and repositioning the expertise of the practitioners and beneficiaries, qualitative paradigms and experimental questions in order to address the interventional system more profoundly.

## Abbreviations

CMOs: Context-mechanism-outcome configurations
COM-B model: Capacity-Opportunity-Motvation – Behaviour Model
EE-TIS: Évaluation de l’Efficacité de l’application Tabac Info Service
GREENH-City: GoveRnance for Equity ENvironment and Health in the City
ICHI: Classification of Health Interventions (ICHI)
MRC: Medical Research Council
TC-REG: Transfert de Connaissances en REGion
TDE: Theory-driven evaluation.
TIS: Tabac Info service
